# Analysis of an Anomaly: The Increase in Time Float following Consumption

**DOI:** 10.1155/2014/415870

**Published:** 2014-08-27

**Authors:** Jianxun Qi, Zhixiong Su

**Affiliations:** ^1^School of Economics and Management, North China Electric Power University, Beijing 102206, China; ^2^Business Administration College, Nanchang Institute of Technology, Nanchang 330099, China

## Abstract

One fundamental axiom for project plan and schedule relates to the notion that time float will be reduced following its consumption. However, an anomalous scenario can emerge in which an activity's time float increases following its consumption. By exploring the associations between time float and paths in activity networks, we (a) reveal the conditions under which the anomaly occurs and (b) summarize laws related to total float. An activity's total float increases in parallel with its duration prolongation within a given boundary but remains constant or decreases in parallel with a prolongation outside the boundary. Furthermore, whereas a prolongation of an activity's duration in excess of classic total float does not delay project completion time, a lag of its start time to a degree slightly greater than the total float does. This analysis reveals different types of total float that correspond to different ways of usage. From this, we offer definitions for translation total float and prolongation total float that deviate from traditional conventions regarding the uniqueness of total float.

## 1. Introduction

Current trends in production are characterized by an increasingly intense competition in sectors dependent on time. George [1] first regarded time as a source of competitive advantage, thereby prompting managers to emphasize the importance of time performance in project management [2–4]. Many researchers have explored time-based competition [5–7], which consists of several time-related concepts. These concepts include processing time, setup time, zero time, and just-in-time delivery [8]. Careful planning is antecedent to the achievement of competitive advantages related to time. Related to this, multiple theories and industry practices have shown that time float is a key factor for developing such a plan [9–14]. An activity's time float not only signifies the degree to which that activity is important to a project but also reflects the project's structural properties and guides project plan and schedule. Given its import, time float has long been considered as an important parameter for project optimization.

Similar to its counterpart in the field of geometry (e.g., the parallel axiom in Euclidean geometry), one fundamental axiom of project plan and schedule stipulates that an activity's time float will be reduced following its consumption. However, we reveal an anomaly that an activity's time float increases rather than decreases while it is being consumed. This contradicts the fundamental axiom of time float. Furthermore, as a source of competitive advantage, an increase in time float following consumption may reflect the possibility that other special sources may increase following consumption. Given these findings, the anomaly may provide valuable insight into resource optimization. Therefore, in this paper, we empirically explore the time float anomaly with the goal of project optimization.

The anomaly described above appears in complex projects, such as projects with generalized precedence relations (GPRs). The GPRs are temporal constraints that mandate that the starting/finishing times of a pair of activities be separated by a minimum or maximum amount of time. This anomaly contradicts not only the time float axiom but also several current approaches to project optimization. For instance, in resource and duration optimization, managers often reduce some resources (e.g., staff, funds, and materials for noncritical activities) to reduce costs or apply resources from noncritical activities to critical activities to expedite project completion. Reductions in resources for noncritical activities are constrained by the activities' total floats. Reducing these resources to a substantial degree can cause prolongation of an activity's duration. Conventional thinking dictates that the prolongation of an activity's duration in excess of its total float delays project completion time. However, the discovery of the “postconsumption total float increase” anomaly seems to free managers from the need to reduce staff, funds, or materials and extends space to allow for resource optimization. However, this anomaly raises several questions.

- Not all activities cause total float to increase. What conditions are necessary for the anomaly to occur?
- Does an activity's total float consistently increase in parallel with the prolongation of its duration? If not, when will total float not increase? How will total float change after the activity duration is prolonged?
- Although an activity's float consumption may cause total float to increase, will its duration prolongation delay project completion time? If yes, by how much?

The answers to these and other questions can significantly influence project plan and schedule.

In this paper, we explain the anomaly and primarily focus on laws related to total floats and the prolongation of activity duration to address the questions above. These conclusions theoretically contribute to discussions related to total float and provide guidance related to project optimization with GPRs.

## 2. Previous Work

Following the emergence of GPRs [15, 16], some authors have studied the characteristics of these precedence relations [17–26]. Others have investigated project scheduling with GPRs, such as resource-constrained project scheduling with GPRs [23, 27–38], time-cost tradeoff with GPRs [18, 22, 39, 40], and resource leveling with GPRs [41–45].

Scholars often extend theoretical premises within their respective fields by discovering theoretical contradictions to conventionally held ideas. For example, extensions within the field of earth science have allowed scientists to observe the curvature of light. Similarly, extensions of the study of activity networks (representing projects) allowed for the discovery of some abnormal characteristics [17–20, 25]. In particular, Elmaghraby and Kamburowski [18] uncovered anomalies related to critical activities (with classic time floats equal to 0) under GPRs.

- The prolongation of a critical activity's duration reduces (rather than increases) the time it takes to complete the project.
- Both the prolongation and shortening of a critical activity's duration left the time to complete the project unchanged.
- Reducing the duration of a critical activity renders the activity network infeasible.

Critical activities are those that are crucial to moving a project towards completion, and difficulty in project plan in general has a significant impact on critical tasks and measures [46]. The discovery of anomalies associated with critical activities shook the conceptual foundations of the classic approaches to project scheduling and provided the basis for many of the approaches that are currently used. One of the anomalies' greatest impacts relates to the time-cost tradeoff with GPRs, because the tradeoff process involves the adjustment of critical activities' durations. Elmaghraby and Kamburowski [18] investigated the time-cost tradeoffs with GPRs by prolonging instead of shortening activity durations. Some others utilized mathematical programming and approximate methods [22, 39, 40]. The adjustment of activity duration is also a method for evaluating resource-constrained project scheduling and resource leveling in such a way to determine how the anomalies influence the problems with GPRs. Some authors have developed other approaches to solve these problems in their investigations of the nature of the anomalies and have included the branch-and-bound algorithm [28–33], the approximate algorithm [42, 43], the subgradient algorithm [32, 33], the min-flow algorithm [35], and the path-relinking metaheuristic algorithm [45]. These approaches effectively dealt with critical activity anomalies and have served as viable contributions to the field of plan management.

Although a substantial amount of empirical attention has been paid to critical activities, extant research on noncritical activities (with time floats greater than 0) under GPRs has been insufficient thus far. To redress this deficiency in the literature, we extend previous research and focus on noncritical activities.

## 3. Generalized Precedence Relations (GPRs)

### 3.1. Types of GPRs

GPRs can be classified as* Finish-to-Start*,* Finish-to-Finish*,* Start-to-Start*, and* Start-to-Finish* relations. These four constraint types double when minimum and maximum time lags are concerned, as follows.

*Finish-to-Start* minimum time lag (FTS_*AB*_^min⁡^(*r*)): the start time of an activity *B* occurs no earlier than *r* units after the finish time of an activity *A*.

*Finish-to-Finish* minimum time lag (FTF_*AB*_^min⁡^(*r*)): the finish time of an activity *B* occurs no earlier than *r* units after the finish time of an activity *A*.

*Start-to-Start* minimum time lag (STS_*AB*_^min⁡^(*r*)): the start time of an activity *B* occurs no earlier than *r* units after the start time of an activity *A*.

*Start-to-Finish* minimum time lag (STF_*AB*_^min⁡^(*r*)): the finish time of an activity *B* occurs no earlier than *r* units after the start time of an activity *A*.

*Finish-to-Start* maximum time lag (FTS_*AB*_^max⁡^(*r*)): the start time of an activity *B* occurs no later than *r* units after the finish time of an activity *A*.

*Finish-to-Finish* maximum time lag (FTF_*AB*_^max⁡^(*r*)): the finish time of an activity *B* occurs no later than *r* units after the finish time of an activity *A*.

*Start-to-Start* maximum time lag (STS_*AB*_^max⁡^(*r*)): the start time of an activity *B* occurs no later than *r* units after the start time of an activity *A*.

*Start-to-Finish* maximum time lag (STF_*AB*_^max⁡^(*r*)): the finish time of an activity *B* occurs no later than *r* units after the start time of an activity *A*.

### 3.2. Representation of GPRs

**Figure 1:**
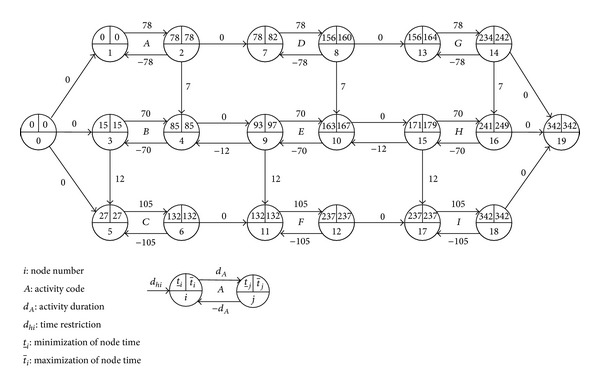
Example of an activity network under GPRs.

The activity network under GPRs proposed by Elmaghraby and Kamburowski [18] is the current standard activity-on-arc representation of GPRs. In the network, an activity is represented as two oppositely directed arcs with opposite length values (whose absolute values are equal to the activity duration). A time lag is represented as an arc.  Table 1 lists activities of a project and precedence relations among them, and they can be represented as a standard activity network under GPRs, as shown in Figure 1.

The following equations depict classic time parameters of an activity *A* = (*i*, *j*), which contain the earliest start time ES_*A*_, latest finish time LF_*A*_, total float TF_*A*_, free float FF_*A*_, and safety float SF_*A*_:

(1) $$\begin{array}{c} {\text{ES}}_{A}={\underline{t}}_{i}=L\left(\mu_{0\to i}^{\nabla }\right), \end{array}$$

(2) $$\begin{array}{c} {\text{LF}}_{A}={\bar{t}}_{j}=L\left(\mu^{\nabla }\right)-L\left(\mu_{j\to n}^{\nabla }\right), \end{array}$$

(3) $$\begin{array}{c} {\text{TF}}_{A}={\bar{t}}_{j}-{\underline{t}}_{i}-d_{A}, \end{array}$$

(4) $$\begin{array}{c} {\text{FF}}_{A}=\underset{i,j}{\min}\underset{(i,k),(j,r)\in P}{\min}\left\{{\underline{t}}_{k}-{\underline{t}}_{i}-d_{ik},{\underline{t}}_{r}-{\underline{t}}_{j}-d_{jr}\right\}, \end{array}$$

(5) $$\begin{array}{c} {\text{SF}}_{A}=\underset{i,j}{\min}\underset{(h,i),(g,j)\in P}{\min}\left\{{\bar{t}}_{i}-{\bar{t}}_{h}-d_{hi},{\bar{t}}_{j}-{\bar{t}}_{g}-d_{gj}\right\}, \end{array}$$

where *μ*_*i*→*j*_^∇^ indicates the longest path from the node (*i*) to node (*j*), *μ* ^∇^ indicates the critical path, and *P* indicates the set of arcs representing time lags.

## 4. Anomaly: The Increase in Time Float following Consumption

Conventional thinking dictates that the total float limits the degree to which an activity's duration can be prolonged and is determined by the structure of the activity network structure (e.g., the preceding and succeeding activities of the activity and project completion). As axioms, it is widely accepted that (a) the prolongation of an activity's duration consumes total float and (b) the degree to which the prolongation exceeds total float delays the project completion time.

However, we have discovered anomalies that contradict these axioms. Specifically, we have found that total float can increase following its consumption and that it is possible for the project completion to avoid delay, even if the prolongation of one of its constituent activities exceeds the total float.

For example, in Figure 1, according to (3), the total float of activity *H* can be computed as follows:

(6) TFH=t¯16−t_15−dH=249−171−70=8.

**Figure 2:**
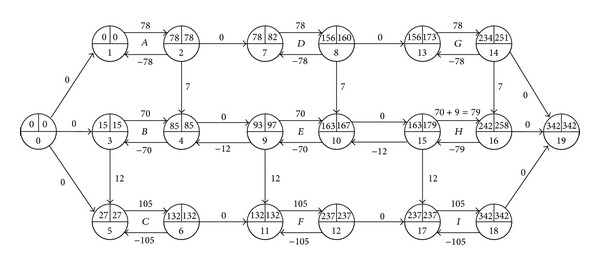
Network with duration prolongation 9 of the activity *H*.

We prolong its duration by 9 (see Figure 2) and compute its total float as follows:

(7) TFH′=t¯16′−t_15′−dH′=257−163−78=16.

Although the durations of other activities and project completion time are unchanged, the prolongation of the duration of activity *H* results in the increase instead of decrease in its total float. Furthermore, the prolongation 9 of activity *H* is greater than its initial total float 8, but it does not cause a delay in the project completion time. The example tests that the total float of an activity may be invalid to restrain the activity's duration.

**Figure 3:**
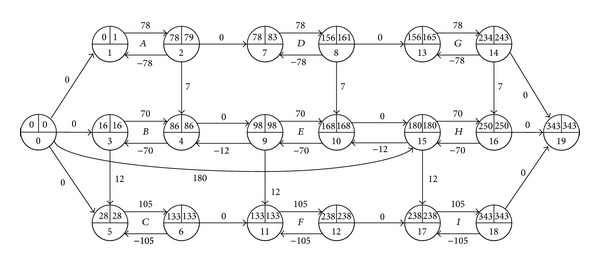
Delaying start time of the activity *H* by 9 in Figure 1.

**Figure 4:**
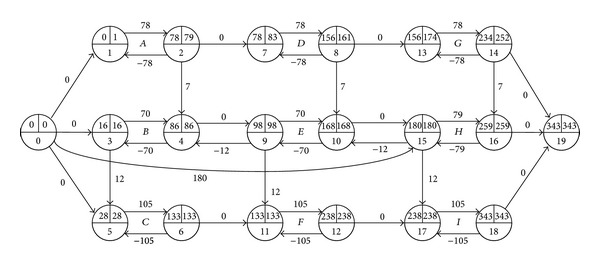
Delaying start time of the activity *H* by 17 in Figure 2.

However, when we delay activity *H*'s start time by 9 in Figure 1 (equal to adding an arc (0,15) with *d*_0,15_ = 180, as in Figure 3), the project completion is delayed by 1. And when we delay activity *H*'s start time by 17 in Figure 2 (its total float is 16 in the figure), as in Figure 4, the project completion is delayed by 1 too. The example illustrates that the total float can restrain the start time of an activity with a given duration, and it may increase following the prolongation of the activity's duration.

The above research signifies that the total float may have different values in accordance with its different usages. We challenge the notion of the classical total float and define two new types of total floats—“translation total float” and “prolongation total float.”

(*1) Translation Total Float.* Under constraints that retain the project completion time, the translation total float is defined as the maximum amount of time that the start of activity *A* with a given duration can be delayed from its earliest start time. We mark it as TTF_*A*_, which is identical to the classical total float TF_*A*_ in nature; that is,

(8) $$\begin{array}{c} {\text{TTF}}_{A}={\text{TF}}_{A}. \end{array}$$

(*2) Prolongation Total Float.* Under constraints that retain the project completion time, the prolongation total float is defined as the maximum amount of time that the duration of activity *A* can be prolonged from its initial one. We mark it as PTF_*A*_, which may be different from TF_*A*_.

The two new types of total floats provide reasonable explanations for the anomaly of the time float increases following its consumption. The translation total float of an activity may increase when the activity prolongs its duration (consumes its prolongation total float). There is no anomaly in the case of considering each type of total float, respectively; that is, the translation (prolongation) total float decreases following its consumption.

## 5. Preliminary Theory

### 5.1. Accessory Concepts

In order to facilitate descriptions in the following sections, here we introduce some parameters: for an arc (*i*, *j*) in the activity network under GPRs,

(9) $$\begin{array}{c} {\text{FF}}_{ij}={\underline{t}}_{j}-{\underline{t}}_{i}-d_{ij}, \end{array}$$

(10) $$\begin{array}{c} {\text{SF}}_{ij}={\bar{t}}_{j}-{\bar{t}}_{i}-d_{ij}, \end{array}$$

(11) $$\begin{array}{c} {\text{TF}}_{ij}={\bar{t}}_{j}-{\underline{t}}_{i}-d_{ij}, \end{array}$$

and for a node (*i*),

(12) FΔFi=min⁡⁡{FFki,(k,i)∈P},

(13) $$\begin{array}{c} {\text{SF}}_{j}^{\Delta }=\min\left\{{\text{SF}}_{jr},\left(j,r\right)\in P\right\}. \end{array}$$

### 5.2. Relations between Parameter and Path

In exploring the relationships between the above parameters and paths, we have deduced the following theorems and corollaries. The relationships are indispensable to uncover laws of time parameters of an activity in the next section.

**Theorem 1 .**

For the longest path *μ*_0→*i*_^∇^ and a path *μ*_0→*i*_ from the beginning node (0) to a node (*i*), their lengths satisfy

(14) $$\begin{array}{c} L\left(\mu_{0\to i}^{\nabla }\right)-L\left(\mu_{0\to i}\right)=\overset{}{\underset{(u,v)\in \mu_{0\to i}}{\sum }}{FF}_{uv}. \end{array}$$

**Proof**

See Appendix A.

**Theorem 2 .**

For the longest path *μ*_*i*→*n*_^∇^ and a path *μ*_*i*→*n*_ from a node (*i*) to the terminal node (*n*), their lengths satisfy

(15) $$\begin{array}{c} L\left(\mu_{i\to n}^{\nabla }\right)-L\left(\mu_{i\to n}\right)=\overset{}{\underset{(u,v)\in \mu_{i\to n}}{\sum }}{SF}_{uv}. \end{array}$$

**Proof**

Similar to Theorem 1.

## 6. Laws of Time Parameters of an Activity

Assume that an activity *A* is represented as two arcs: arc(*i*, *j*) with length *d*_*A*_ and arc(*j*, *i*) with length −*d*_*A*_. Activity *A*'s start and finish nodes are (*i*) and (*j*), respectively. In this paper, we explore laws of time parameters associated with activity *A*.

The activity network under GPRs permits no cycles with positive length. If arc(*i*, *j*) is on a cycle (Φ_*ij*_) with length *L*(Φ_*ij*_) ≤ 0, a prolongation of activity *A* such that Δ*d*_*A*_ > |*L*(Φ_*ij*_)| will result in an infeasible network with *L*(Φ_*ij*_′) > 0. For the purposes of our investigation, we assume *L*(Φ_*ij*_) ≪ 0 to ensure that Δ*d*_*A*_ ≤ |*L*(Φ_*ij*_)|.

### 6.1. The Earliest Start Time

In conventional approaches to exploring float, the earliest start time of an activity is determined by its preceding activities. Therefore, an activity's earliest start time will remain unchanged as long as other activities remain unchanged as well.

However, we find that an activity's start time may be advanced even if other activities remain unchanged (see Figure 2). In this subsection, we study this anomaly and summarize the following law of the earliest start time of an activity *A* associated with this anomaly. For the activity *A*, assume that ES_*A*_ and ES_*A*_′ denote its initial and following earliest start times, and *d*_*A*_ and *d*_*A*_′ denote its initial and following durations, respectively.

**Law 1 .**

If ^Δ^FF_*i*_ > 0, the laws of the earliest start time ES_*A*_ are as follows when *d*_*A*_′ = *d*_*A*_ + Δ*d*_*A*_ and Δ*d*_*A*_ > 0.

(1) If 0 < Δ*d*_*A*_ ≤  ^Δ^FF_*i*_, then

(16) $$\begin{array}{c} {\text{ES}}_{A}^{\prime }={\text{ES}}_{A}-\Delta d_{A} \end{array}$$

and then

(17) $$\begin{array}{c} d_{A}^{\prime }↑⟹{\text{ES}}_{A}^{\prime }↓. \end{array}$$

(2) If Δ*d*_*A*_ >  ^Δ^FF_*i*_, then

(18) ESA′=ESA−FΔFi.

This reflects the notion that the actual earliest start time is earlier than the classical earliest start time ES_*A*_.

**Proof**

See Appendix B.

### 6.2. The Latest Finish Time

Conventional thinking dictates that the latest finish time of an activity is determined by the activities that succeed it and the overall completion of the project. As such, a latest finish time will not be delayed as long as the durations of other activities and project completion time remain unchanged.

However, we find that the latest finish time may be delayed even under the above conditions (see Figure 2). By exploring this anomaly, we summarize the following law of the latest finish time of an activity *A* associated with this anomaly. For the activity *A*, assume that LF_*A*_ and LF_*A*_′ denote its initial and following latest finish times, respectively.

**Law 2 .**

If SF_*j*_^Δ^ > 0, under the condition of retaining project completion time, the laws of the latest finish time LF_*A*_ are as follows when *d*_*A*_′ = *d*_*A*_ + Δ*d*_*A*_ and Δ*d*_*A*_ > 0.

(1) If 0 < Δ*d*_*A*_ ≤ SF_*j*_^Δ^, then

(19) $$\begin{array}{c} {\text{LF}}_{A}^{\prime }={\text{LF}}_{A}+\Delta d_{A} \end{array}$$

and then

(20) $$\begin{array}{c} d_{A}^{\prime }↑⟹{\text{LF}}_{A}^{\prime }↑. \end{array}$$

(2) If Δ*d*_*A*_ > SF_*j*_^Δ^, then

(21) $$\begin{array}{c} {\text{LF}}_{A}^{\prime }={\text{LF}}_{A}+{\text{SF}}_{j}^{\Delta }. \end{array}$$

This demonstrates that the actual latest finish time is later than the classical latest finish time *LF*_*A*_.

**Proof**

See Appendix C.

### 6.3. Total Float

**Figure 5:**
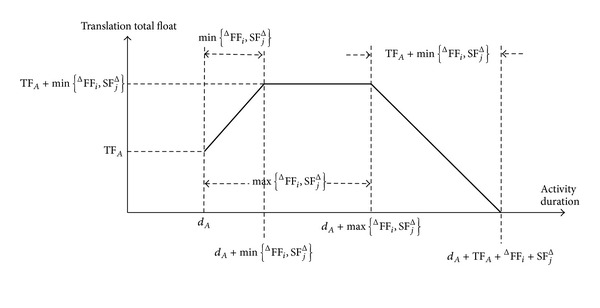
Curve chart of the translation total float when  ^Δ^FF_*i*_ > 0 and SF_*j*_^Δ^ > 0.

**Figure 6:**
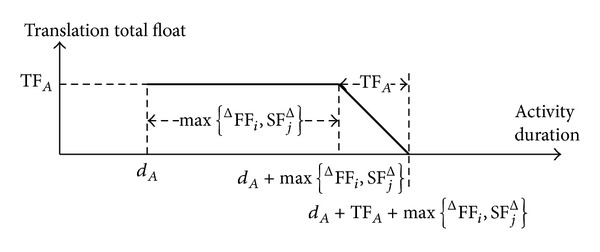
Curve chart of the translation total float of the activity *A* when ^Δ^FF_*i*_ or SF_*j*_^Δ^ (but not both) is equal to 0.

**Figure 7:**
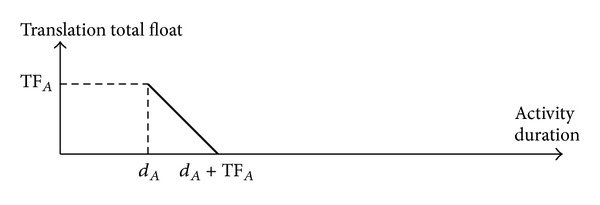
Curve chart of the translation total float of the activity *A* when ^Δ^FF_*i*_ = 0 and SF_*j*_^Δ^ = 0.

According to Section 4, the translation (prolongation) total float decreases following its consumption, but the translation total float of an activity may increase when the activity consumes its prolongation total float. We summarize the following laws of the total float of an activity *A* (as in Figures 5–7). For the activity *A*, assume that TTF_*A*_ and TTF_*A*_′ denote its initial and following translation total floats, respectively, and denote its initial classical total float.

**Law 3 .**

If ^Δ^FF_*i*_ > 0 and SF_*j*_^Δ^ > 0, then for *d*_*A*_′ = *d*_*A*_ + Δ*d*_*A*_ and Δ*d*_*A*_ > 0 we have the following.

(1) If 0 < Δ*d*_*A*_ ≤ min⁡⁡{ ^Δ^FF_*i*_, SF_*j*_^Δ^}, then

(22) $$\begin{array}{c} {\text{TTF}}_{A}^{\prime }={\text{TF}}_{A}+\Delta d_{A}={\text{TTF}}_{A}+\Delta d_{A} \end{array}$$

and then

(23) $$\begin{array}{c} d_{A}^{\prime }↑⟹{\text{TTF}}_{A}^{\prime }↑ \end{array}$$

(2) If min⁡⁡{^Δ^FF_*i*_, SF_*j*_^Δ^} < Δ*d*_*A*_ ≤ max⁡⁡{ ^Δ^FF_*i*_, SF_*j*_^Δ^}, then

(24) TTFA′=TFA+min⁡⁡{FΔFi,SFjΔ}

and then

(25) $$\begin{array}{c} d_{A}^{\prime }↑⟹{\text{TTF}}_{A}^{\prime }\;\;\left(\text{unchanged}\right). \end{array}$$

(3) If max⁡⁡{^Δ^FF_*i*_, SF_*j*_^Δ^} < Δ*d*_*A*_ ≤ TF_*A*_ + ^Δ^FF_*i*_ + SF_*j*_^Δ^, then

(26) TTFA′=TFA+FΔFi+SFjΔ−ΔdA

and then

(27) $$\begin{array}{c} d_{A}^{\prime }↑⟹{\text{TTF}}_{A}^{\prime }↓. \end{array}$$

(4) If Δ*d*_*A*_ > TF_*A*_ + ^Δ^FF_*i*_ + SF_*j*_^Δ^, then the project completion time will be delayed. This shows that the prolongation total float needed to maintain the project completion is greater than the classical one TF_*A*_.

**Proof**

See Appendix D.

**Law 4 .**

If ^Δ^FF_*i*_ or SF_*j*_^Δ^ (but not both) is equal to 0, then for *d*_*A*_′ = *d*_*A*_ + Δ*d*_*A*_ and Δ*d*_*A*_ > 0 we have the following.

(1) If 0 < Δ*d*_*A*_ ≤ max⁡⁡{ ^Δ^FF_*i*_, SF_*j*_^Δ^}, then

(28) $$\begin{array}{c} {\text{TTF}}_{A}^{\prime }={\text{TF}}_{A}={\text{TTF}}_{A} \end{array}$$

and then

(29) $$\begin{array}{c} d_{A}^{\prime }↑⟹{\text{TTF}}_{A}^{\prime }\;\;\left(\text{unchanged}\right). \end{array}$$

(2) If max⁡⁡{ ^Δ^FF_*i*_, SF_*j*_^Δ^} < Δ*d*_*A*_ ≤ TF_*A*_ + max⁡⁡{^Δ^FF_*i*_, SF_*j*_^Δ^}, then

(30) TFA′=TFA−ΔdA+max⁡⁡{FΔFi,SFjΔ}

and then

(31) $$\begin{array}{c} d_{A}^{\prime }↑⟹{\text{TTF}}_{A}^{\prime }↓. \end{array}$$

(3) If Δ*d*_*A*_ > TF_*A*_ + max⁡⁡{^Δ^FF_*i*_, SF_*j*_^Δ^}, the project completion will be delayed. This reflects that the actual total float needed to maintain the project completion exceeds the classic one TF_*A*_.

**Proof**

See Appendix E.

**Law 5 .**

If ^Δ^FF_*i*_ = 0 and SF_*j*_^Δ^ = 0, then

(32) $$\begin{array}{c} {\text{TTF}}_{A}^{\prime }={\text{TF}}_{A}-\Delta d_{A}={\text{TTF}}_{A}-\Delta d_{A} \end{array}$$

and then

(33) $$\begin{array}{c} d_{A}^{\prime }↑⟹{\text{TTF}}_{A}^{\prime }↓ \end{array}$$

when *d*_*A*_′ = *d*_*A*_ + Δ*d*_*A*_ and 0 < Δ*d*_*A*_ ≤ TF_*A*_.

**Proof**

See Appendix F.

According to Laws 3–5, we achieve the following sufficient and necessary conditions of the anomaly in Section 4 and deduce the formula of prolongation total float.

**Corollary 3 .**

For an activity *A*, ^Δ^ *FF*_*i*_ > 0 and *SF*_*j*_^Δ^ > 0 are sufficient and necessary conditions for total float to increase following consumption. Moreover, the duration interval for this anomaly is [*d*_*A*_, *d*_*A*_ + min⁡⁡{^Δ^ *FF*_*i*_, *SF*_*j*_^Δ^}].

**Corollary 4 .**

The prolongation total float of activity *A* is

(34) PTFA=min⁡⁡{TFA+FΔFi+SFjΔ,|L(Φij)|}

and *L*(Φ_*ij*_) denotes the lengths of cycles Φ_*ij*_ passing the arc(*i*, *j*) but not passing the arc(*j*, *i*).

## 7. A Numerical Example

To illustrate the arguments outlined above, we analyze the laws of total float of activity *H* in Figure 1.

According to Figure 1 and (9)–(13), *H* is represented as arcs (15,16) and (16,15), and

(35) FΔF15=min⁡{FF10,15}=t_15−t_10−d10,15=171−163−0=8,SF16Δ=min⁡⁡{SF16,19}=t¯19−t¯16−d16,19=342−249−0=93,TFH=t¯16−t_15−dH=249−171−70=8.

Because ^Δ^FF_15_ > 0, SF_16_^Δ^ > 0, and no cycles pass the arc (15,16), according to Law 3 in Section 6.3.

(1) If 0 < Δ*d*_*H*_ ≤ min⁡⁡{^Δ^FF_15_, SF_16_^Δ^}, namely, 0 < Δ*d*_*H*_ ≤ 8 and *d*_*H*_′ ∈ (70,78], then

(36) $$\begin{array}{c} {\text{TTF}}_{H}^{\prime }={\text{TF}}_{H}+\Delta d_{H}={\text{TTF}}_{H}+\Delta d_{H} \end{array}$$

and then

(37) $$\begin{array}{c} d_{H}^{\prime }↑⟹{\text{TTF}}_{H}^{\prime }↑. \end{array}$$

(2) If min⁡⁡{^Δ^FF_15_, SF_16_^Δ^} < Δ*d*_*H*_ ≤ max⁡⁡{^Δ^FF_15_, SF_16_^Δ^}, namely, 8 < Δ*d*_*H*_ ≤ 93 and *d*_*H*_′ ∈ (78,163], then

(38) TTFH′=TFH+min⁡⁡{FΔF15,SF16Δ}=8+min⁡⁡{8,93}=16

and then

(39) $$\begin{array}{c} d_{H}^{\prime }↑⟹{\text{TTF}}_{H}^{\prime }\;\;\left(\text{unchanged}\right). \end{array}$$

(3) If max⁡⁡{^Δ^F F_15_, SF_16_^Δ^} < Δ*d*_*H*_ ≤ TF_*H*_ + ^Δ^FF_15_ + SF_16_^Δ^, namely, 93 < Δ*d*_*H*_ ≤ 109 and *d*_*H*_′ ∈ (163,179], then

(40) TTFH′=TFH+FΔF15+SF16Δ−ΔdH=8+8+93−ΔdH=109−ΔdH

and then

(41) $$\begin{array}{c} d_{H}^{\prime }↑⟹{\text{TTF}}_{H}^{\prime }↓. \end{array}$$

(4) If Δ*d* > TF_*H*_ + ^Δ^FF_15_ + SF_16_^Δ^, namely, Δ*d*_*H*_ > 109 and *d*_*H*_′ ∈ (109, +*∞*), the project completion will be delayed.

**Figure 8:**
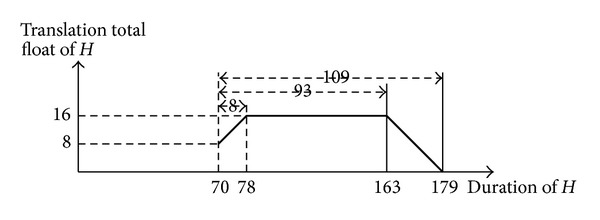
Curve chart of translation total float of the activity *H*.

**Figure 9:**
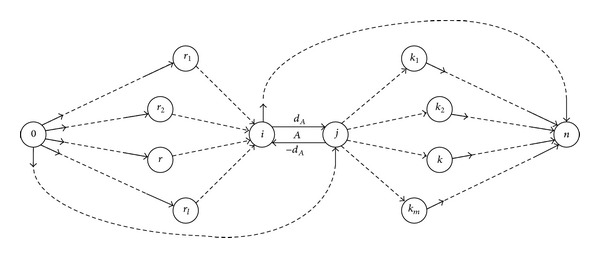
Diagram of paths passing the activity *A* in an activity network under GPRs.

We summarize these results as a variety of laws related to translation total float of activity *H* in the process of consuming its prolongation total float. In doing so, we obtain Figure 8. For activity *H*, we have the following.

- In the duration interval [70,78], its translation total float increases (from 8 to 16) in conjunction with the duration prolongation.
- In the duration interval (78,163], its translation total float remains unchanged (the value is 16) despite the duration prolongation.
- In the duration interval (163,179], its translation total float decreases (from 16 to 0) in conjunction with the duration prolongation.
- Its duration 179 causes the translation total float to be equal to 0, causing a delay of its start time to induce a delay in the project completion.

According to (34), because Φ_15,16_ is nonexistent, we let |*L*(Φ_15,16_)| = +*∞* and compute

(42) PTFH′=min⁡{TFH+FΔF15+SF16Δ,|L(Φ15,16)|}=TFH+FΔF15+SF16Δ=109.

Therefore, in the duration interval [70,179] of *H*, its prolongation total float consistently decreases (from 109 to 0) in conjunction with the duration prolongation. As a result, delaying project completion requires a longer time than its duration of 179. In addition, because TTF_*H*_ = 8, the project completion time will be delayed if (a) activity *H*'s start time is delayed by more than 8 or (b) its duration is prolonged more than 109.

Furthermore, we test that the translation total floats of activities *B* and *E* also increase following the prolongation of their durations. But the ones of the other activities are bound to decrease following the prolongation of their durations.

## 8. Conclusions

The phenomenon whereby time float increases following its consumption is a perfect condition for planning and scheduling that may simplify various problems and optimize operations. However, conventional theorizing about float time suggests that this phenomenon is imaginary. The view that time float is reduced when it is consumed remains a dominant perspective for project plan and schedule.

Challenging established axioms often results in crucial scientific advances. For example, non-Euclidean geometry was created on the basis of challenges to the parallel axiom in Euclidean geometry. Similarly, we questioned the generally accepted time float axiom and discovered the existence of an anomaly such that time float increases following consumption. Time is regarded as a source of competitive advantage; therefore, the anomaly of time float provides the prospect that other sources may also increase following consumption. We gain an enlightenment from the anomaly that the total float may be manifold corresponding to different usages and uncover two new types: translation total float and prolongation total float. The two concepts illustrate the multivalued nature of total float, which challenges conventional theorizing about the uniqueness of total float. We further summarize the laws of total floats, which perfectly explain the anomaly.

Despite its contributions, this paper does not consider other activity time floats, such as free float and safety float. Our future research endeavors include the study of laws related to these time floats following their respective consumption and the application of those laws to optimize project plan and schedule.

## Figures and Tables

**Table 1:** Precedence relations between activities.

Activity	Duration	Immediate successor	Time lag
*A*	78	*B*, *D*	FTF_*AB*_^min⁡^(7), FTS_*AD*_^min⁡^(0)
*B*	70	*C*, *E*	STS_*BC*_^min⁡^(12), FTS_*BE*_^min⁡^(0), FTS_*BE*_^max⁡^(12)
*C*	105	*F*	FTS_*CF*_^min⁡^(0)
*D*	78	*E*, *G*	FTF_*DE*_^min⁡^(7), FTS_*DG*_^min⁡^(0)
*E*	70	*F*, *H*	STS_*EF*_^min⁡^(12), FTS_*EH*_^min⁡^(0), FTS_*EH*_^max⁡^(12)
*F*	105	*I*	FTS_*FI*_^min⁡^(0)
*G*	78	*H*	FTF_*GH*_^min⁡^(7)
*H*	70	*I*	STS_*HI*_^min⁡^(12)
*I*	105		

## Appendices

### A. Proof of Theorem 1

Assume *μ*_0→*i*_ = (0) → (*a*) → (*b*) → ⋯→(*r*)→(*i*), according to (9),

(A.1) ∑(u,v)∈μ0→iFFuv=FF0a+FFab+···+FFri=(t_a−t_0−d0a)+(t_b−t_a−dab) +···+(t_i−t_r−dri)=t_i−t_0−d0a−dab−···−dri=t_i−t_0−L(μ0→i).

And according to (1),

(A.2) $$\begin{array}{c} {\underline{t}}_{0}=0,\;{\underline{t}}_{i}=L\left(\mu_{0\to i}^{\nabla }\right). \end{array}$$

Therefore,

(A.3) $$\begin{array}{c} \underset{(u,v)\in \mu_{0\to i}}{\sum }{\text{FF}}_{uv}=L\left(\mu_{0\to i}^{\nabla }\right)-L\left(\mu_{0\to i}\right). \end{array}$$

Equation (14) is correct.

### B. Proof of Law 1

Figure 9 shows each type of path passing activity *A* in an activity network under GPRs. According to (11) and (14), assume ^Δ^FF_*i*_ = FF_*ri*_; then *L*(*μ*_0→*ri*_^∇^) ≥ *L*(*μ*_0→*r*_*x*_*i*_^∇^), *x* = 1,2,…, *l*. And according to Figure 9,

(B.1) $$\begin{array}{c} L\left(\mu_{0\to i}^{\nabla }\right)=\max\left\{L\left(\mu_{0\to ri}^{\nabla }\right),L\left(\mu_{0\to ji}^{\nabla }\right)\right\}. \end{array}$$

Substituting (1) in (B.1),

(B.2) $$\begin{array}{c} {\text{ES}}_{A}=\max\left\{L\left(\mu_{0\to ri}^{\nabla }\right),L\left(\mu_{0\to ji}^{\nabla }\right)\right\}. \end{array}$$

Owing to FF_*ri*_ = ^Δ^FF_*i*_ > 0, according to (14), *L*(*μ*_0→*i*_^∇^) − *L*(*μ*_0→*ri*_^∇^) = FF_*ri*_ > 0 so that

(B.3) $$\begin{array}{c} L\left(\mu_{0\to i}^{\nabla }\right)>L\left(\mu_{0\to ri}^{\nabla }\right). \end{array}$$

According to (B.1),

(B.4) $$\begin{array}{c} L\left(\mu_{0\to i}^{\nabla }\right)=L\left(\mu_{0\to ji}^{\nabla }\right), \end{array}$$

and substitute it in (14) as follows

(B.5) $$\begin{array}{c} L\left(\mu_{0\to ji}^{\nabla }\right)-L\left(\mu_{0\to ri}^{\nabla }\right)={\text{FF}}_{ri}. \end{array}$$

Therefore,

(B.6) $$\begin{array}{c} L\left(\mu_{0\to ji}^{\nabla }\right)=L\left(\mu_{0\to ri}^{\nabla }\right)+{\text{FF}}_{ri}. \end{array}$$

In Figure 9, the arcs (*i*, *j*) and (*j*, *i*) are not on *μ*_0→*ri*_^∇^ and *μ*_0→*j*_^∇^. If *d*_*A*_′ = *d*_*A*_ + Δ*d*_*A*_ for Δ*d*_*A*_ > 0, then *d*_*ij*_′ = *d*_*ij*_ + Δ*d*_*A*_ and

(B.7) L(μ0→ri∇′)=L(μ0→ri∇),L(μ0→j∇′)=L(μ0→j∇).

*d*_*ji*_ = *d*_*A*_ causes *d*_*ji*_′ = *d*_*ji*_ − Δ*d*_*A*_; therefore,

(B.8) L(μ0→ji∇′)=L(μ0→j∇′)+dji′=L(μ0→j∇)+dji−ΔdA=L(μ0→ji∇)−ΔdA;

that is,

(B.9) $$\begin{array}{c} L\left(\mu_{0\to ji}^{\nabla^{\prime }}\right)=L\left(\mu_{0\to ji}^{\nabla }\right)-\Delta d_{A}. \end{array}$$

According to (1) and (B.2)–(B.9),

(B.10) ESA′=max⁡⁡{L(μ0→ri∇′),L(μ0→ji∇′)}=max⁡⁡{L(μ0→ri∇),L(μ0→ji∇)−ΔdA}=max⁡⁡{L(μ0→ri∇),L(μ0→ri∇)+FFri−ΔdA}=max⁡⁡{L(μ0→ji∇)−FFri,L(μ0→ji∇)−ΔdA}=max⁡{L(μ0→i∇)−FFri,L(μ0→i∇)−ΔdA}=max⁡⁡{ESA−  FΔFi,ESA−ΔdA}.

Therefore, if Δ*d*_*A*_ ≤  ^Δ^FF_*i*_, then

(B.11) $$\begin{array}{c} {\text{ES}}_{A}^{\prime }={\text{ES}}_{A}-\Delta d_{A}. \end{array}$$

If Δ*d*_*A*_ >  ^Δ^FF_*i*_, then

(B.12) ESA′=ESA−FΔFi.

Equations (16) and (18) are correct.

### C. Proof of Law 2

According to (13) and (14), assume SF_*j*_^Δ^ = SF_*jk*_ (see Figure 9); then *L*(*μ*_*jk*→*n*_^∇^) ≥ *L*(*μ*_*jk*_*y*_→*n*_^∇^), *y* = 1,2,…, *m*. Based on Figure 9,

(C.1) $$\begin{array}{c} L\left(\mu_{j\to n}^{\nabla }\right)=\max\left\{L\left(\mu_{jk\to n}^{\nabla }\right),L\left(\mu_{ji\to n}^{\nabla }\right)\right\}. \end{array}$$

Substituting it in (2),

(C.2) $$\begin{array}{c} {\text{LF}}_{A}=L\left(\mu^{\nabla }\right)-\max\left\{L\left(\mu_{jk\to n}^{\nabla }\right),L\left(\mu_{ji\to n}^{\nabla }\right)\right\}. \end{array}$$

Owing to SF_*jk*_ = SF_*j*_^Δ^ > 0, according to (15), *L*(*μ*_*j*→*n*_^∇^) − *L*(*μ*_*jk*→*n*_^∇^) = SF_*jk*_ > 0 so that

(C.3) $$\begin{array}{c} L\left(\mu_{j\to n}^{\nabla }\right)>L\left(\mu_{jk\to n}^{\nabla }\right). \end{array}$$

According to (C.1),

(C.4) $$\begin{array}{c} L\left(\mu_{j\to n}^{\nabla }\right)=L\left(\mu_{ji\to n}^{\nabla }\right). \end{array}$$

combined with (15),

(C.5) $$\begin{array}{c} L\left(\mu_{ji\to n}^{\nabla }\right)-L\left(\mu_{jk\to n}^{\nabla }\right)={\text{SF}}_{jk}. \end{array}$$

In Figure 9, the arcs (*i*, *j*) and (*j*, *i*) are not on *μ*_*jk*→*n*_^∇^ and *μ*_*i*→*n*_^∇^. If *d*_*A*_′ = *d*_*A*_ + Δ*d*_*A*_ for Δ*d*_*A*_ > 0, then *d*_*ij*_′ = *d*_*ij*_ + Δ*d*_*A*_, *d*_*ji*_′ = *d*_*ji*_ − Δ*d*_*A*_, and

(C.6) L(μi→n∇′)=L(μi→n∇),L(μjk→n∇′)=L(μjk→n∇).

Therefore,

(C.7) L(μji→n∇′)=dji′+L(μi→n∇′)=dji−ΔdA+L(μi→n∇)=dji+L(μi→n∇)−ΔdA=L(μji→n∇)−ΔdA;

that is,

(C.8) $$\begin{array}{c} L\left(\mu_{ji\to n}^{\nabla \prime }\right)=L\left(\mu_{ji\to n}^{\nabla }\right)-\Delta d_{A}. \end{array}$$

According to the known condition, *L*(*μ* ^∇′^) = *L*(*μ* ^∇^). Therefore, combining it with (2) and (C.2)–(C.8),

(C.9) LFA′=L(μ∇′)−max⁡⁡{L(μjk→n∇′),L(μji→n∇′)}=L(μ∇)−max⁡⁡{L(μjk→n∇),L(μji→n∇)−ΔdA}=L(μ∇)−max⁡⁡{L(μji→n∇)−SFjk,L(μji→n∇)−ΔdA}=L(μ∇)−max⁡⁡{L(μj→n∇)−SFjΔ,L(μ0→j∇)−ΔdA}=min⁡⁡{L(μ∇)−L(μj→n∇)+SFjΔ,L(μ∇)−L(μj→n∇)+ΔdA}=max⁡⁡{LFA+SFjΔ,LFA+ΔdA}.

Therefore, if Δ*d*_*A*_ ≤ SF_*j*_^Δ^, then

(C.10) $$\begin{array}{c} {\text{LF}}_{A}^{\prime }={\text{LF}}_{A}+\Delta d_{A}. \end{array}$$

Further, if Δ*d*_*A*_ > SF_*j*_^Δ^, then

(C.11) $$\begin{array}{c} {\text{LF}}_{A}^{\prime }={\text{LF}}_{A}+{\text{SF}}_{j}^{\Delta }. \end{array}$$

Equations (19) and (21) are correct.

### D. Proof of Law 3

We assume that 0 < ^Δ^FF_*i*_ ≤ SF_*j*_^Δ^. Therefore, min⁡{^Δ^FF_*i*_, SF_*j*_^Δ^} = ^Δ^FF_*i*_ and max⁡⁡{^Δ^FF_*i*_, SF_*j*_^Δ^} = SF_*j*_^Δ^. Assume *d*_*A*_′ = *d*_*A*_ + Δ*d*_*A*_ and Δ*d*_*A*_ > 0.

(1) If 0 < Δ*d*_*A*_ ≤ min⁡⁡{^Δ^FF_*i*_, SF_*j*_^Δ^}, namely, 0 < Δ*d*_*A*_ ≤ ^Δ^FF_*i*_ ≤ SF_*j*_^Δ^, according to (1), (2), (16), and (19), ${\underline{t}}_{i}^{\prime }={\underline{t}}_{i}-\Delta d_{A}$, ${\bar{t}}_{j}^{\prime }={\bar{t}}_{j}+\Delta d_{A}$. Substituting them in (3),

(D.1) TFA′=t¯j′−t_i′−dA′=(t¯j+ΔdA)−(t_i−ΔdA)−(dA+ΔdA)=(t¯j−t_i−dA)+ΔdA=TFA+ΔdA.

Equation (22) is correct.

(2) If min⁡⁡{^Δ^FF_*i*_, SF_*j*_^Δ^} < Δ*d*_*A*_ ≤ max⁡⁡{^Δ^FF_*i*_, SF_*j*_^Δ^}, namely, ^Δ^FF_*i*_ < Δ*d*_*A*_ ≤ SF_*j*_^Δ^, according to (1), (2), (18), and (21), t_i′=t_i-FΔFi,  ${\bar{t}}_{j}^{\prime }={\bar{t}}_{j}+\Delta d_{A}$. Substituting them in (3),

(D.2) TFA′=t¯j′−t_i′−dA′=(t¯j+ΔdA)−(t_i−FΔFi)−(dA+ΔdA)=(t¯j−t_i−dA)+FΔFi=TFA+FΔFi.

Equation (24) is correct.

(3) If Δ*d*_*A*_ > max⁡⁡{^Δ^FF_*i*_, SF_*j*_^Δ^}⁡, namely, Δ*d*_*A*_ > SF_*j*_^Δ^ ≥ ^Δ^FF_*i*_, according to (1), (2), (18), and (21),  t_i′=t_i-FΔFi,  ${\bar{t}}_{j}^{\prime }={\bar{t}}_{j}+{\text{SF}}_{j}^{\Delta }$. Substituting them in (3),

(D.3) TFA′=t¯j′−t_i′−dA′=(t¯j+SFjΔ)−(t_i−FΔFi)−(dA+ΔdA)=(t¯j−t_i−dA)+FΔFi+SFjΔ−ΔdA=TFA+FΔFi+SFjΔ−ΔdA.

Under the precondition of retaining project completion, TF_*A*_′ ≥ 0, hence

(D.4) TFA+FΔFi+SFjΔ−ΔdA≥0 ⟹ΔdA≤TFA+FΔFi+SFjΔ.

Equation (26) is correct.

(4) If Δ*d*_*A*_ = ^Δ^FF_*i*_ + TF_*A*_ + SF_*j*_^Δ^, according to (3) and (11), TF_*A*_′ = TF_*ij*_′ = 0 and activity *A* becomes a critical one. Now a new critical path *μ* ^∇^ passes the arc(*i*, *j*). If Δ*d*_*A*_ > ^Δ^FF_*i*_ + TF_*A*_ + SF_*j*_^Δ^, the prolongation of *d*_*ij*_ will cause a prolongation in *μ* ^∇^. Therefore, the project completion will be delayed, and the actual total float for retaining the project completion time is TF_*A*_ + ^Δ^FF_*i*_ + SF_*j*_^Δ^ > TF_*A*_.

### E. Proof of Law 4

In Figure 5, if either ^Δ^FF_*i*_ or SF_*j*_^Δ^ (but not both) is equal to 0, then

(E.1) min⁡⁡{FΔFi,SFjΔ}=0,TFA+min⁡⁡{FΔFi,SFjΔ}=TFA,dA+min⁡⁡{FΔFi,SFjΔ}=dA,dA+TFA+FΔFi+SFjΔ=dA+TFA+max⁡⁡{FΔFi,SFjΔ}.

Therefore, Figure 5 becomes Figure 6, and Figure 6 illustrates Law 4.

### F. Proof of Law 5

In Figure 6, if ^Δ^FF_*i*_ = 0 and SF_*j*_^Δ^ = 0, then

(F.1) max⁡⁡{FΔFi,SFjΔ}=0,dA+max⁡⁡{FΔFi,SFjΔ}=dA,dA+TFA+FΔFi+SFjΔ=dA+TFA.

Therefore, Figure 6 becomes Figure 7, and Figure 7 illustrates Law 5.
